# Age- and sex-related differences in the retinal capillary plexus in healthy Chinese adults

**DOI:** 10.1186/s40662-022-00307-0

**Published:** 2022-10-01

**Authors:** Binbin Su, Xiaoxuan Zhu, Kai Yang, Yunfan Xiao, Chunmei Li, Keai Shi, Jia Qu, Fan Lu, Ming Li, Lele Cui

**Affiliations:** Eye Hospital and School of Ophthalmology and Optometry, Wenzhou Medical University, National Clinical Research Center for Ocular Diseases, Wenzhou, Zhejiang 325003 People’s Republic of China

**Keywords:** Retinal capillary plexus, Aging, Sex-related, Optical coherence tomography angiography, Vessel density

## Abstract

**Background:**

To assess age- and sex-related changes in the superficial retinal capillary plexus (SCP) and deep retinal capillary plexus (DCP) in healthy Chinese adults.

**Methods:**

In this cross-sectional study, all data were derived from the community-based Jidong Eye Cohort Study. Participants underwent optical coherence tomography angiography (OCTA) and other ocular and systemic examinations. The vessel densities of the whole measured area, parafovea, and four quadrants in the SCP and DCP were measured.

**Results:**

We recruited 1036 eyes of 1036 healthy participants; the mean age was 40.4 ± 9.8 years, and 449 (43.3%) participants were males. The SCP and DCP vessel densities in all regions, except for temporal and nasal regions in the SCP, non-linearly decreased with age. The DCP vessel densities began to decrease at approximately 35 years of age, while the SCP vessel densities began to decrease at approximately 40 years of age. The DCP vessel densities decreased more rapidly than the SCP vessel densities at 35–50 years of age. The DCP vessel densities remained stable or slightly decreased after the age of 50 years in females, while those decreased linearly in most regions in males.

**Conclusions:**

The retinal vessel density decreased earlier and more rapidly in the DCP than in the SCP, and the effect of aging on the DCP vessel density was sex-dependent. Our findings suggest that age and sex should be considered when interpreting clinical quantitative OCTA data.

**Supplementary Information:**

The online version contains supplementary material available at 10.1186/s40662-022-00307-0.

## Background

The retina, one of the most metabolically active tissues in the body, has a unique vascular system to meet the high oxygen demand required for metabolism [1]. A sound retinal vascular system is essential for maintaining normal retinal metabolism and visual function, and retinal diseases can be diagnosed or predicted based on its evaluation. Optical coherence tomography angiography (OCTA) is a new non-invasive fundus imaging technique for high-resolution retinal microcirculation assessment [2–4]. The retinal capillary system can be divided into three major plexuses using OCTA: the superficial retinal capillary plexus (SCP), the intermediate retinal capillary plexus, and the deep retinal capillary plexus (DCP) [5]. However, due to projection artifacts, the retinal capillary plexus is commonly separated into only the SCP and DCP. The vessel densities from these two areas decrease to different extents in diseases such as age-related macular degeneration and diabetic retinopathy [6–8].

Like other organs, the retinal vascular system changes with age [9]. Thus, it is critical to distinguish age-related physiological changes from pathological alterations of the retinal vascular system. Some studies have shown that aging is negatively associated with vessel density of the whole measured area and/or parafovea in the SCP and DCP [5, 10–12], but others have found that the association is not significant [13–15]. It remains unknown whether the relationship between the change in the retinal microvasculature and aging is linear. Additionally, some studies indicate sex differences in age-related cardiovascular and cerebrovascular changes [16, 17]. Some studies found that men had higher SCP vessel density than women [11, 18]; however, others have not found this [13]. Therefore, we aimed to determine whether the changes in the patterns of SCP and DCP vessel densities are age-dependent. Further, we explored whether the male or female sex plays a role in these changes. To help us answer these, we quantified vascular macular variables in healthy eyes and investigated their relationship with age and sex.

## Methods

### Study design and participants

The data for this study were derived from the Jidong Eye Cohort Study (JECS), a population-based multipurpose study conducted in the Jidong communities of Tangshan, Northern China. The details of the study have been published previously [19]. The study was approved by the Ethics Committee of Staff Hospital of the Jidong Oil-Field of Chinese National Petroleum (China National Petroleum Corporation Jidong Oil-Field Branch Staff Hospital approval document of the medical ethics committee, No. 2018 YILUNZI 1) and was performed in accordance with the tenets of the Declaration of Helsinki. All participants provided written informed consent.

Data were collected from 3377 participants who underwent a comprehensive examination between August 2019 and January 2020. Inclusion criteria were age between 20 and 80 years and axial length (AL) between 23 and 25 mm. Participants were excluded if they had systemic pathologies such as hypertension, diabetes, cerebrovascular diseases, cardiovascular diseases, cancer, autoimmune diseases; ocular diseases; pathological changes on optical coherence tomography (OCT) scans; poor-quality OCTA scan images; or best-corrected visual acuity worse than 20/25. One eye from each participant was included in the analysis. The right eye was selected if both eyes met the criteria for the study.

### Assessment of general variables

Participants were interviewed and completed a structured questionnaire on demographic characteristics, smoking habits, alcohol consumption, and medical history. The height and weight of each participant were measured, and body mass index (BMI) was calculated. Blood pressure was measured using an automatic digital blood pressure monitor. Mean arterial pressure (MAP) was calculated as one third systolic blood pressure plus two thirds diastolic blood pressure. Blood samples were obtained by venipuncture to determine fasting blood glucose. Hypertension was defined as having systolic blood pressure ≥ 140 mmHg or diastolic blood pressure ≥ 90 mmHg. Diabetes was defined as having a fasting glucose concentration of ≥ 126 mg/dl. Self-reported history of hypertension or diabetes medication use, and diagnostic history of hypertension or diabetes were also recorded.

### Ophthalmic examination

The ophthalmic examination included visual acuity testing with a standard logarithmic visual acuity chart, non-cycloplegic objective refraction with an autorefractor (Topcon KR-800; Topcon Tokyo, Japan), and subjective refraction with trial frames to obtain the best-corrected visual acuity. Measurements of AL and central corneal thickness (CCT) were obtained using the Lenstar LS 900 (Haag-Streit, Koeniz, Switzerland). The anterior ocular segments were examined using a slit-lamp microscope, and the posterior ocular segments were examined using a fundus camera (Canon CR-2AF; Canon, Tokyo, Japan) and a spectral-domain OCT device (AngioVue; Optovue, Fremont, CA, USA).

**Fig. 1:**
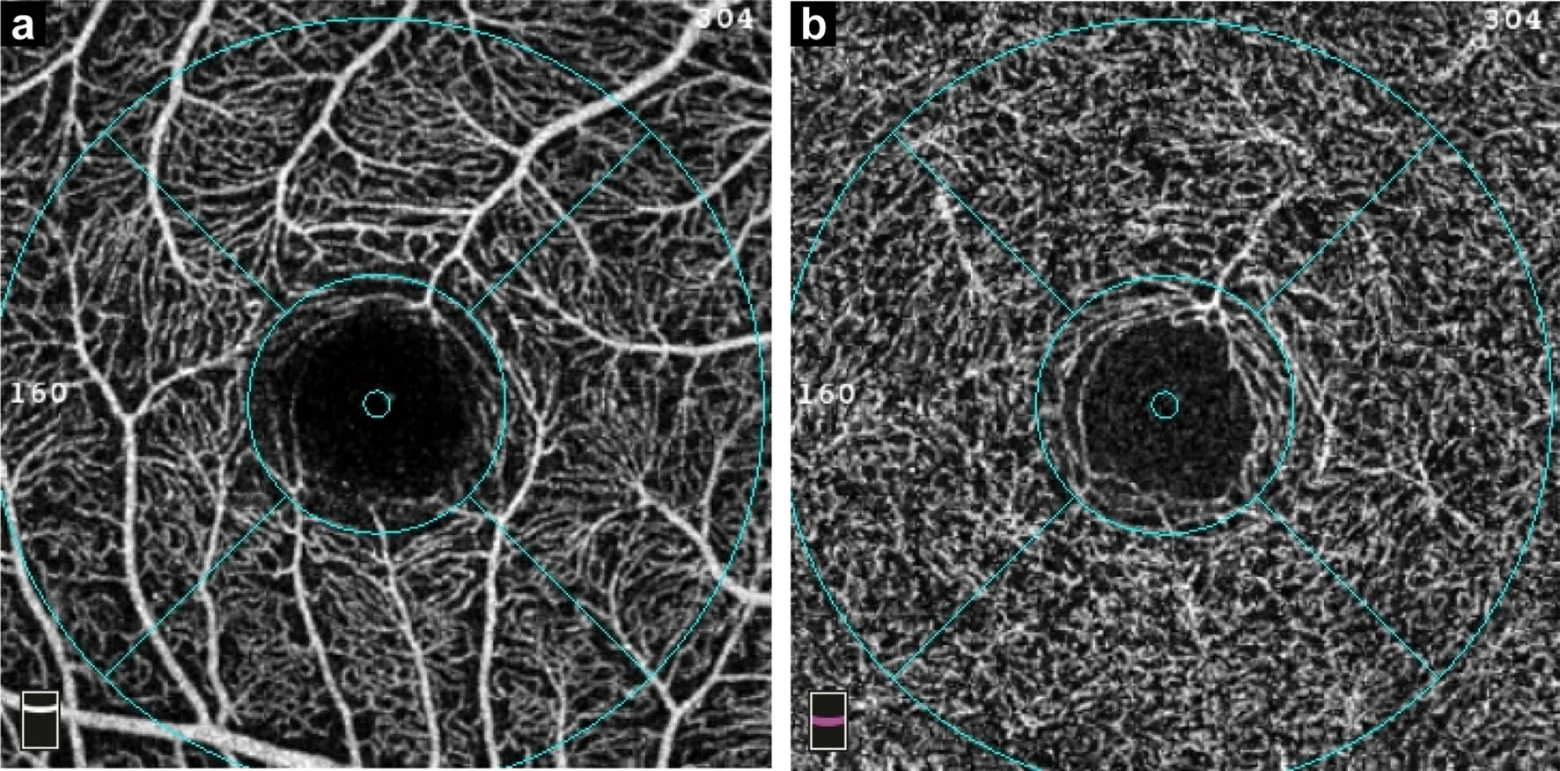
Macular vessel density in the fovea, parafovea, and four quadrants (temporal, superior, nasal and inferior) were calculated based on the ETDRS contour. **a** Superficial retinal capillary plexus; **b** Deep retinal capillary plexus. ETDRS, Early Treatment Diabetic Retinopathy Study

OCTA and OCT images were acquired using a spectral-domain OCT device. OCTA images of the macula, centered on the fovea, were acquired using the 3 × 3 mm scan with a scan density of 304 × 304 A-scans. Scan images with poor quality such as signal strength index (SSI) < 7, obvious motion artifacts, poor centering, or incorrect segmentations were excluded. The vessel density of the SCP and DCP was measured automatically by the software (version 2017.1.0.155) in the device. The projection artifacts in the DCP were removed by the device’s in-built 3D projection artifacts removal algorithms. Vessel density was defined as the percentage of the vessel area with blood flow in the sample area. The SCP was set from the internal limiting membrane to 9 μm above the lower boundary of the inner plexiform layer. The DCP was set from 9 μm above the lower boundary of the inner plexiform layer to 9 μm below the lower boundary of the outer plexiform layer. Vessel density was automatically generated by the instrument based on the 3 mm partial Early Treatment Diabetic Retinopathy Study grid (Fig. 1). The measured area was the whole 3 mm circular area centered on the fovea. The parafovea was the area of the 1–3 mm concentric rings at the center of the fovea; it was further divided into four quadrants (temporal, superior, nasal, and inferior).

### Statistical analysis

Participants were divided into five age groups, i.e., 20–29 years, 30–39 years, 40–49 years, 50–59 years, and 60 years or older. Continuous variables, shown as mean ± standard deviation (SD), were analyzed using t-tests or one-way analysis of variance. Categorical variables, expressed as counts (percentages), were analyzed using the χ^2^ test or Fisher’s exact test, as appropriate.

Restricted cubic splines with four knots (5th, 35th, 65th, and 95th centiles) were used to flexibly model the association of age with angiographic parameters after adjusting for sex, education, current smoking status, current drinking status, MAP, BMI, AL, and SSI. The same association analysis was also performed in a population stratified by sex. Multivariate generalized linear regression analysis was used to determine the association of age with angiographic parameters. The β coefficients were reported with 95% confidence intervals (CIs). A *P* value of less than 0.05 was considered statistically significant. Statistical analyses were performed using SPSS 25.0 (IBM, Inc., Chicago, IL, USA) and R 4.1.0 (https://www.R-project.org).

## Results

### Baseline characteristics of eligible participants

**Fig. 2:**
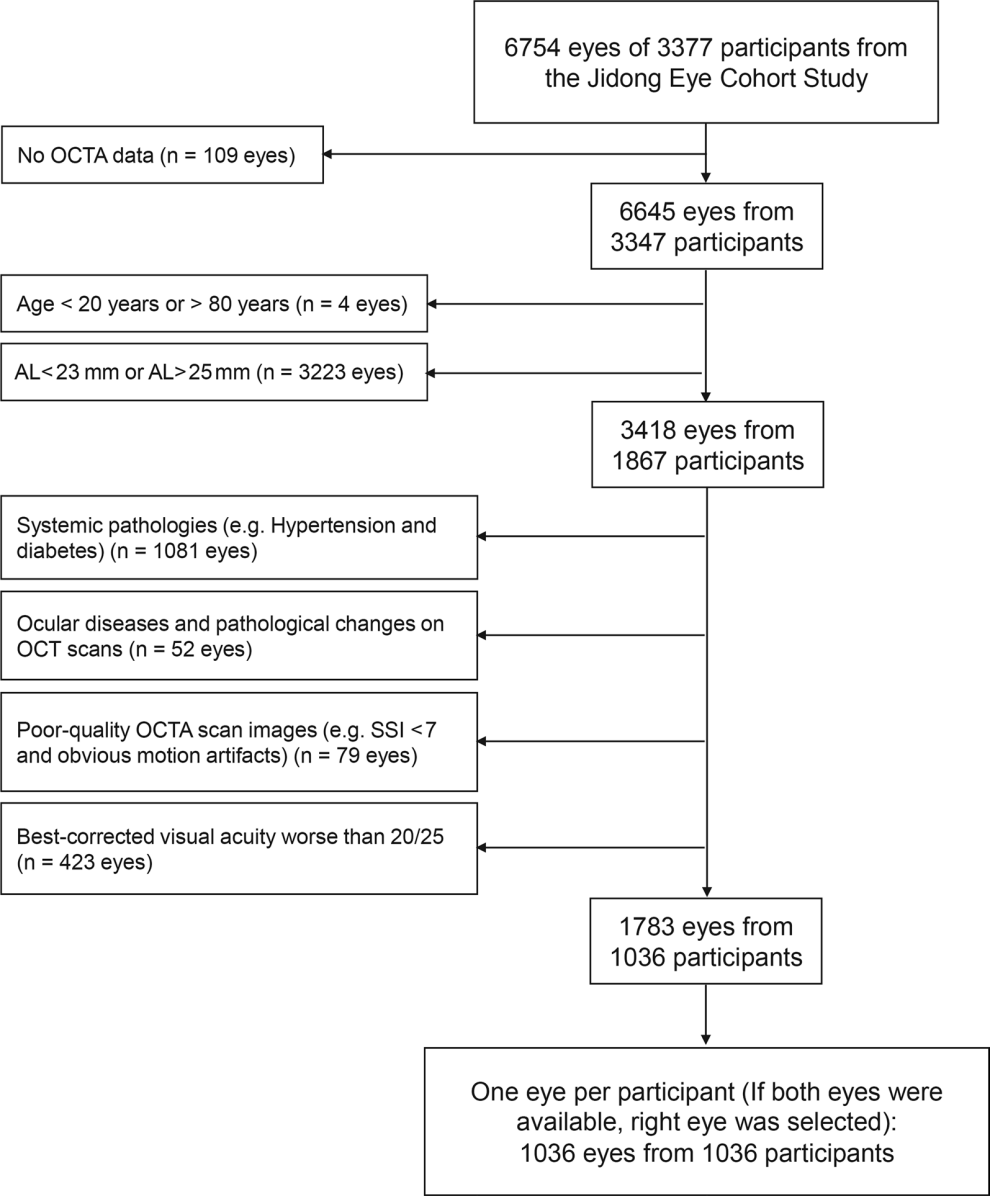
Inclusion and exclusion criteria of study eyes. AL, axial length; SSI, signal strength index of optical coherence tomography angiography image

Of the 3347 JECS participants (n = 6645 eyes) with OCTA images, 1036 participants (n = 1783 eyes) met our inclusion and exclusion criteria. Given that one eye per participant was imaged, a total of 1036 eyes from 1036 participants were analyzed (Fig. 2).

Table 1 shows the demographic, systemic, and ocular characteristics of the 1036 included participants based on age. The mean age was 40.4 ± 9.8 years (range, 22–79 years), and 449 (43.3%) participants were males. Compared with their younger counterparts, older participants had a lower educational level, shorter AL, higher MAP, higher spherical equivalent refraction, and lower SSI. In addition, there were statistically significant differences in the current smoking and drinking status among different age groups of males (see Additional file 1: Table S1), and remarkable differences in BMI among different age groups of females (see Additional file 1: Table S2).

**Table 1:** Baseline characteristics of eligible participants in the study

	Total (n = 1036)	Age group (years)					*P*
		20–29	30–39	40–49	50–59	≥ 60	
		(n = 79)	(n = 516)	(n = 240)	(n = 153)	(n = 48)	
Age (mean ± SD, years)	40.4 ± 9.8	27.0 ± 1.9	34.4 ± 2.6	44.3 ± 2.9	53.3 ± 2.6	65.2 ± 4.1	< 0.001
Male, n (%)	449 (43.3%)	32 (40.5%)	229 (44.4%)	90 (37.5%)	70 (45.8%)	28 (58.3%)	0.07
Education level, n (%)							< 0.001
Middle school and below	214 (20.7)	5 (6.3)	35 (6.8)	68 (28.3)	70 (45.8)	36 (75.0)	
College/University	813 (78.5)	74 (93.7)	473 (91.7)	172 (71.7)	82 (53.6)	12 (25.0)	
Current smoking, n (%)	202 (19.5)	16 (10.3)	96 (18.6)	52 (21.7)	31 (20.3)	7 (14.6)	0.79
Current drinking, n (%)	124 (12.0)	6 (7.6)	53 (10.3)	38 (15.8)	22 (14.4)	5 (10.4)	0.12
BMI (kg/m^2^)	23.8 ± 3.3	23.3 ± 4.2	23.6 ± 3.4	24.1 ± 3.3	23.8 ± 2.7	24.0 ± 2.6	0.18
MAP (mmHg)	87.5 ± 8.9	87.1 ± 8.3	86.2 ± 8.6	88.8 ± 9.1	89.2 ± 9.4	90.0 ± 9.0	< 0.001
SE (D)	− 1.5 ± 1.7	− 1.7 ± 1.5	− 1.9 ± 1.6	− 1.3 ± 1.6	− 0.5 ± 1.4	0.5 ± 1.1	< 0.001
AL (mm)	24.0 ± 0.6	24.1 ± 0.5	24.1 ± 0.6	23.9 ± 0.6	23.8 ± 0.6	23.7 ± 0.5	< 0.001
SSI	8.6 ± 0.6	8.6 ± 0.7	8.6 ± 0.6	8.6 ± 0.6	8.6 ± 0.6	8.3 ± 0.8	0.004

*SD* = standard deviation; *BMI* = body mass index; *MAP* = mean arterial pressure; *SE* = spherical equivalent refraction; *D* = diopter; *AL* = axial length; *SSI* = signal strength index of optical coherence tomography angiography image

### Vessel density by age groups

**Fig. 3:**
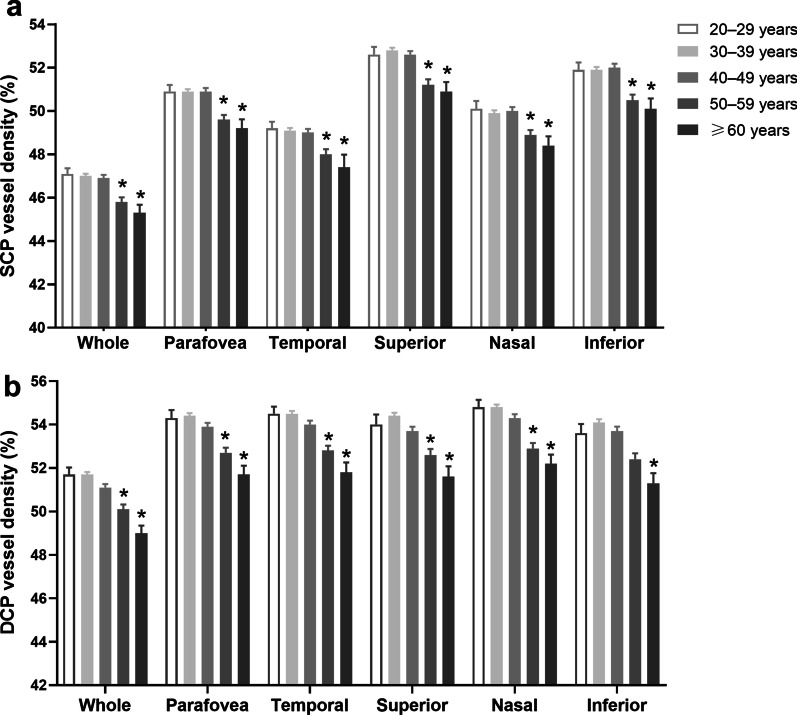
The vessel densities in the SCP (**a**) and DCP (**b**) measured with OCTA and stratified by age. Data are shown as mean ± standard error. *Indicates statistical difference compared with the 20- to 29-year age group (*P* < 0.05). SCP, superficial retinal capillary plexus; DCP, deep retinal capillary plexus; OCTA, optical coherence tomography angiography

In both the SCP and DCP, the mean vessel densities began to decrease significantly in almost all regions at 50 to 59 years (Fig. 3). Vessel densities of the whole measured area, parafovea, and four quadrants in the SCP and DCP stratified by age and sex are shown in Additional file 1: Table S3. The trend of mean vessel density change with age was not consistent between males and females (see Additional file 1: Fig. S1).

### Associations of each age group with the vessel density

Table 2 shows the association of age with vessel density in the whole population after adjusting for confounders. In the SCP, compared to the 30- to 39-year age group, the 50- to 59-year age group had a significantly lower vessel density in the whole measured area (β, − 0.96; 95% CI, − 1.41 to − 0.50), parafovea (β, − 1.07; 95% CI, − 1.56 to − 0.58), and four quadrants (Table 2). Significant associations were also found in all investigated regions, except for the nasal and inferior regions of the ≥ 60-year age group. In the DCP, compared to the 30- to 39-year age group, the 40- to 49-year age group showed a significantly lower vessel density in the whole measured area (β, − 0.73; 95% CI, − 1.13 to − 0.33), parafovea (β, − 0.72; 95% CI, − 1.14 to − 0.30), and four quadrants (Table 2). Significant associations for vessel density were also found for the 50- to 59-year and the ≥ 60-year age groups.

**Table 2:** Association of age with vessel density (multivariable analysis) of the whole study population

	Age group (years)									
	20–29		30–39	40–49		50–59		≥ 60		*P*^*b*^
	Adjusted β (95% CI)	*P*^*a*^		Adjusted β (95% CI)	*P*^*a*^	Adjusted β (95% CI)	*P*^*a*^	Adjusted β (95% CI)	*P*^*a*^	
SCP										
Whole	0.07 (− 0.50 to 0.64)	0.81	Ref.	− 0.03 (− 0.40 to 0.35)	0.90	− 0.96 (− 1.41 to − 0.50)	< 0.001	− 0.95 (− 1.72 to − 0.18)	0.02	< 0.001
Parafovea	− 0.03 (− 0.63 to 0.58)	0.94	Ref.	0.00 (− 0.40 to 0.40)	1.00	− 1.07 (− 1.56 to − 0.58)	< 0.001	− 0.87 (− 1.68 to − 0.05)	0.04	< 0.001
Temporal	0.01 (− 0.66 to 0.67)	0.98	Ref.	− 0.09 (− 0.53 to 0.36)	0.70	− 0.94 (− 1.47 to − 0.40)	< 0.001	− 1.03 (− 1.94 to − 0.12)	0.03	0.005
Superior	− 0.27 (− 0.93 to 0.39)	0.42	Ref.	− 0.13 (− 0.58 to 0.31)	0.56	− 1.36 (− 1.90 to − 0.82)	< 0.001	− 1.05 (− 1.95 to − 0.15)	0.02	< 0.001
Nasal	0.13 (− 0.55 to 0.81)	0.70	Ref.	0.11 (− 0.34 to 0.57)	0.63	− 0.81 (− 1.37 to − 0.26)	< 0.001	− 0.71 (− 1.63 to 0.22)	0.13	0.02
Inferior	0.02 (− 0.68 to 0.71)	0.97	Ref.	0.11 (− 0.35 to 0.58)	0.64	− 1.14 (− 1.71 to − 0.58)	< 0.001	− 0.73 (− 1.68 to 0.22)	0.13	0.001
DCP										
Whole	− 0.24 (− 0.83 to 0.36)	0.43	Ref.	− 0.73 (− 1.13 to − 0.33)	< 0.001	− 1.45 (− 1.94 to − 0.97)	< 0.001	− 1.82 (− 2.63 to − 1.02)	< 0.001	< 0.001
Parafovea	− 0.37 (− 1.00 to 0.25)	0.25	Ref.	− 0.72 (− 1.14 to − 0.30)	< 0.001	− 1.58 (− 2.09 to − 1.07)	< 0.001	− 1.73 (− 2.58 to − 0.88)	< 0.001	< 0.001
Temporal	− 0.31 (− 0.93 to 0.32)	0.34	Ref.	− 0.70 (− 0.12 to − 0.28)	< 0.001	− 1.48 (− 1.99 to − 0.98)	< 0.001	− 1.76 (− 2.61 to − 0.90)	< 0.001	< 0.001
Superior	− 0.39 (− 1.13 to 0.36)	0.31	Ref.	− 0.92 (− 1.42 to − 0.43)	< 0.001	− 1.64 (− 2.25 to − 1.04)	< 0.001	− 1.84 (− 2.85 to − 0.83)	< 0.001	< 0.001
Nasal	− 0.18 (− 0.81 to 0.46)	0.59	Ref.	− 0.60 (− 1.03 to − 0.18)	0.01	− 1.69 (− 2.20 to − 1.17)	< 0.001	− 1.77 (− 2.63 to − 0.91)	< 0.001	< 0.001
Inferior	− 0.62 (− 1.36 to 0.12)	0.10	Ref.	− 0.65 (− 1.14 to − 0.16)	0.01	− 1.47 (− 2.06 to − 0.87)	< 0.001	− 1.59 (− 2.59 to − 0.59)	< 0.001	< 0.001

*CI* = confidence interval; *SCP* = superficial retinal capillary plexus; *DCP* = deep retinal capillary plexus; *Ref.* = reference. Adjusted for sex, education, current smoking status, current drinking status, body mass index, mean arterial pressure, axial length, and signal strength index of optical coherence tomography angiography image. ^a^*P* values of each age group compared with the 30- to –39-year age group. ^b^*P* values derived using multivariate generalized linear regression analysis

### Restricted cubic spline analysis for age-related change in vessel density in the whole population

**Fig. 4:**
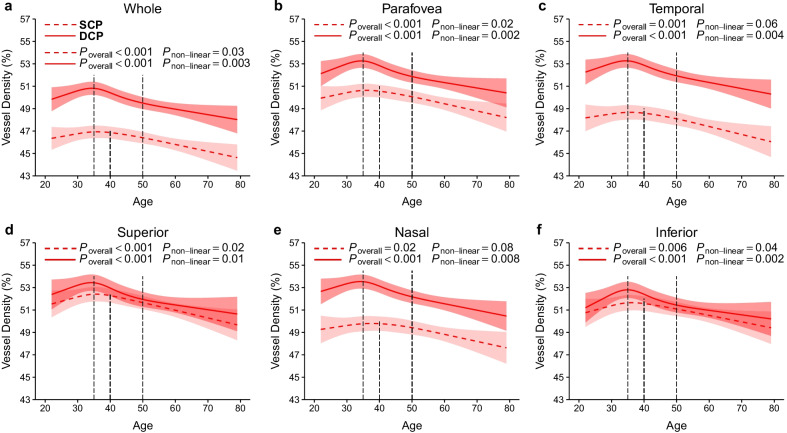
Association of age with vessel densities in the retinal capillary plexus in the whole study population. **a** Whole measure area; **b** Parafovea; **c** Temporal; **d** Superior; **e** Nasal; **f** Inferior. In the SCP (dashed line) and DCP (solid line), the vessel densities decreased non-linearly with age. There was a rapid decline in vessel density at the age of 35–50 years in the DCP compared with the SCP. Models were adjusted for sex, education, current smoking status, current drinking status, body mass index, axial length, and signal strength index of optical coherence tomography angiography image. SCP, superficial retinal capillary plexus; DCP, deep retinal capillary plexus

To visualize the relationship between age and vessel density, we used restricted cubic splines to characterize how vessel density in the SCP and DCP varies with age in the whole population (Fig. 4). In the SCP, the vessel densities in all regions, except for temporal and nasal regions, non-linearly decreased with age (all non-linear *P* < 0.05). Vessel densities in most regions were stable or slightly increased with aging until they began to decrease at the age of 40 years. In the DCP, vessel densities in all regions non-linearly decreased with aging (all non-linear *P* < 0.05). Vessel densities in all regions slightly increased before the age of 35 years but began to decrease after the age of 35 years. In addition, at the age of 35 to 50 years, the DCP vessel densities decreased more rapidly than the SCP vessel densities.

### Restricted cubic spline analysis for age-related changes in vessel density in the population stratified by sex

**Fig. 5:**
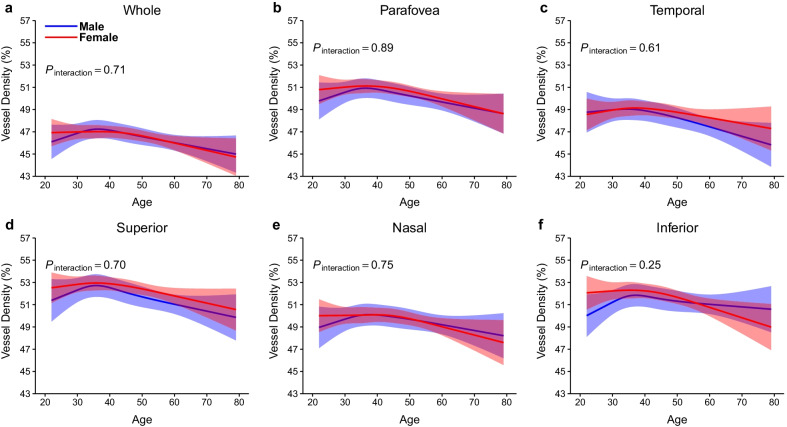
Association of age with vessel densities in the SCP stratified by sex. **a** Whole measure area; **b** Parafovea; **c** Temporal; **d** Superior; **e** Nasal; **f** Inferior. In males (blue band), the SCP vessel densities in most regions decreased linearly with age. In females (red band), the SCP vessel densities of parafovea and inferior regions decreased linearly with age. Models were adjusted for sex, education, current smoking status, current drinking status, body mass index, axial length, and signal strength index of optical coherence tomography angiography image. SCP, superficial retinal capillary plexus

In the SCP, vessel densities of male participants decreased linearly with age in all regions (*P* < 0.05), except for the nasal (*P* = 0.21) and inferior (*P* = 0.17) regions. Among female participants, vessel densities decreased linearly with age only in the parafovea (*P* = 0.03) and inferior (*P* = 0.008) regions. There were no age-by-sex interactions in changes in SCP with aging (all *P* > 0.20; Fig. 5).

**Fig. 6:**
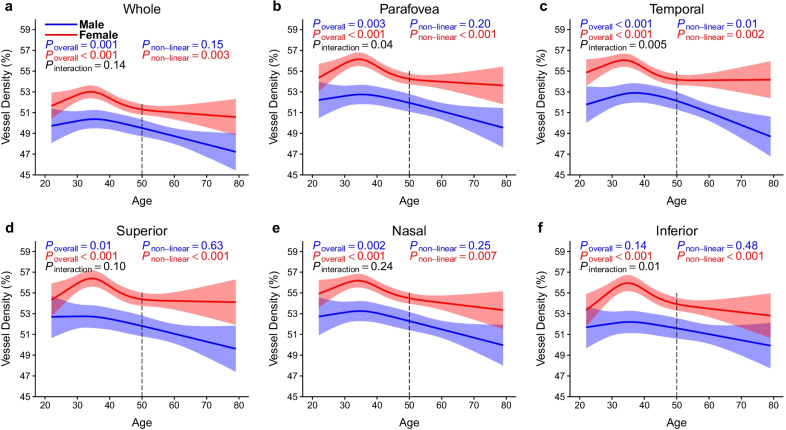
Association of age with vessel densities in the DCP stratified by sex. **a** Whole measure area; **b** Parafovea; **c** Temporal; **d** Superior; **e** Nasal; **f** Inferior. The DCP vessel densities in most regions decreased linearly with age in males (blue band). On the other hand, the DCP vessel densities in all regions decreased non-linearly with age and remained stable or slightly declined after 50 years in females (red band). Models were adjusted for sex, education, current smoking status, current drinking status, body mass index, axial length, and signal strength index of optical coherence tomography angiography image. DCP, deep retinal capillary plexus

In the DCP, vessel densities of female participants showed a non-linear decline with age in all regions (all non-linear *P* < 0.05). The vessel densities showed no significant change or a slight decrease after the age of 50 years. However, the vessel densities of male participants declined linearly with age in all regions (*P* < 0.05), except for the inferior regions (*P* = 0.14). Age-by-sex interactions were significant in the parafovea, temporal, and inferior quadrants in the DCP (*P* = 0.04, *P* = 0.005, and *P* = 0.01, respectively; Fig. 6).

## Discussion

The current study demonstrated that the change patterns of the SCP and DCP vessel densities markedly differed by age and sex. We found that the vessel density in both the SCP and DCP decreased non-linearly with age; the DCP vessel density began to decrease significantly at approximately 35 years of age, which was earlier than the SCP (~ 40 years of age). Moreover, the vessel density in the DCP declined more rapidly at 35–50 years of age when compared with the SCP. Interestingly, the DCP vessel density decreased non-linearly with age in females but linearly with age in males. Notably, the DCP vessel density in females remained stable or slightly decreased after 50 years of age.

Our results show that in the whole population, unadjusted SCP and DCP vessel densities do not change significantly between 20 and 50 years of age but began to decrease after. This finding is consistent with the results of previous studies [13, 18]. However, after adjusting for confounding factors such as sex, AL, and SSI, we demonstrated that the critical age for the significant decrease of vessel density in the DCP was earlier than that in the SCP (Table 2). Therefore, we hypothesize that the trend of decreasing retinal vessel density with age is non-linear and that the changes in SCP and DCP are inconsistent. The restricted cubic spline function further revealed the non-linear variation of retinal capillary plexus vessel density with age. The SCP vessel density remained stable or slightly increased with age until it began to decline at about 40 years of age, while the DCP vessel density increased with age and began to decline at about 35 years of age. Although there was an upward trend in retinal capillary plexus vessel density before the age of 35–40 years, the difference was small and not statistically significant. This is consistent with the results from Abay et al.’s study [20]. Age-related decrease in retinal capillary plexus vessel density may be related to age-related retinal capillary occlusion and atrophy [21]. Compared with the SCP, the DCP is more vulnerable to aging because of the long distance from this plexus to the larger arterioles, higher capillary density, smaller loop area and vessel gap, and higher metabolic demand [22–24]. In addition, Borrelli et al. [25] reported that DCP vessel density was strictly dependent on retinal thickness, which also has been shown to become thinner with age [26, 27].

If we used the 30- to 39-year age group as a reference, the average whole and parafovea vessel density in the SCP decreased by 1.21% and 1.11% per decade and in the DCP decreased by 1.74% and 1.65% per decade after 40 years of age. Further, we demonstrated that the decline rate of vessel density in the DCP was higher compared with the SCP at 35–50 years of age, but the decline rate was similar after 50 years of age. Previous studies found that the average annual decline rate of vessel density in the DCP was higher than that observed in the SCP [5, 10], while other studies did not find any differences [12, 28]. This may be because the results of the average annual or decadal average decline rate could have masked the difference in the rate of decrease of vessel density. The reason why the DCP vessel density decreases more rapidly between the ages of 35 and 50 is unclear. We speculate that at this age, the human body is about to enter or is entering middle age, and senescence accelerates [29, 30]. Because of its histological characteristics, vessel density in the DCP decreases more rapidly than that in the SCP. This may explain why the critical age for the significant decline of vessel density in the DCP was earlier than that found in the SCP.

Our study shows that sex had impacted DCP vessel density with respect to age. In the DCP, vessel densities in females were higher than those in males. Consistent with our results, Wang et al. [11] reported that a higher deep capillary network was associated with the female sex. Notably, hormone therapy has been reported to be beneficial in postmenopausal women with ocular vascular diseases [31, 32], suggesting that retinal vessels are protected by estrogen but damaged by androgens [33]. Our results showed that DCP vessel densities in females increased before the age of 35 years probably with a high level of estrogen, then rapidly decreased probably due to decreasing levels of estrogen, and remained stable or only slightly decreased after the age of 50 years with relatively low level of estrogen. DCP vessel densities in males decreased linearly with age perhaps due to effects of androgens or other non-measured factors. Differences in sex hormones might account for higher DCP in women in each area and age group when compared with men. Additionally, we should not ignore the effects of menopause on SCP and DCP in post-menopausal women. However, sex differences in retinal capillaries and their age-related changes need to be validated further, and the potential mechanism underlying this phenomenon should be explored in future studies.

The large sample size, comprehensive vessel density regions assessed, and adjustment for associated demographic, systemic, and ocular factors, enabled us to analyze age- and sex-related changes in the retinal capillary plexus. However, our study also has some limitations. First, as the design of our study was cross-sectional in nature, our results require further validation by longitudinal studies. Second, this study included a healthy population from only one center. Therefore, our study population may not adequately represent the entire Chinese population and generalization of the findings to other ethnicities would be limited. Third, we did not correct the magnification of the image before the analysis. To reduce the influence of magnification, this study only included eyes with AL in the range of 23–25 mm and selected the parafovea vessel density that was less affected by the magnification as the primary outcome [34]. Statistical analyses were also adjusted for AL as a confounding factor to minimize the impact of magnification.

## Conclusions

In conclusion, we demonstrate here that vessel densities in the SCP and DCP declined with age in a non-linear manner. The DCP vessel density began to decrease significantly at about 35 years of age, which was earlier when compared with the SCP (~ 40 years of age). Compared with the SCP, the vessel density in the DCP decreased more rapidly at 35–50 years of age. The DCP vessel density also remained stable or slightly decreased after 50 years of age in females. Therefore, age and sex should be considered when analyzing macular vessel density in diseased eyes.

## Supplementary Information

**Additional file 1: Figure S1.** Vessel densities of the whole measured area, parafovea, and four quadrants (temporal, superior, nasal, and inferior) in the (**a, c**) SCP and (**b, d**) DCP measured with optical coherence tomography angiography stratified by age in males and females. **Table S1. **Baseline characteristics of male participants in the study. **Table S2.** Baseline characteristics of female participants in the study. **Table S3.** Vessel densities of the whole measured area, parafovea, and four quadrants in the SCP and DCP stratified by age and sex.

## Data Availability

The datasets used and analyzed during the current study are available from the corresponding authors upon reasonable request.

## Abbreviations

SCP: Superficial retinal capillary plexus
DCP: Deep retinal capillary plexus
OCTA: Optical coherence tomography angiography
JECS: Jidong Eye Cohort Study
AL: Axial length
OCT: Optical coherence tomography
BMI: Body mass index
MAP: Mean arterial pressure
CCT: Central corneal thickness
SSI: Signal strength index
SD: Standard deviation
CI: Confidence interval
