# Supplementary material for: Age- and sex-related differences in the retinal capillary plexus in healthy Chinese adults
Source: Eye Vis (Lond). 2022 Oct 1;9:38. doi: 10.1186/s40662-022-00307-0 (PMC9526290; doi:10.1186/s40662-022-00307-0)
Supplement: Supplementary file 1 — Additional file 1: Figure S1. Vessel densities of the whole measured area, parafovea, and four quadrants (temporal, superior, nasal, and inferior) in the (a, c) SCP and (b, d) DCP measured with optical coherence tomography angiography stratified by age in males and females. Table S1. Baseline characteristics of male participants in the study. Table S2. Baseline characteristics of female participants in the study. Table S3. Vessel densities of the whole measured area, parafovea, and four quadrants in the SCP and DCP stratified by age and sex. [file 40662_2022_307_MOESM1_ESM.docx]

**Supplemental Figure 1.** Vessel densities of the whole measured area, parafovea, and four quadrants (temporal, superior, nasal, and inferior) in the **(a, c)** SCP and **(b, d)** DCP measured with optical coherence tomography angiography stratified by age in males and females. Data are shown as mean ±standard error. * Indicates statistical difference compared with the 20- to 29-year age group (*P* < 0.05). SCP, superficial retinal capillary plexus; DCP, deep retinal capillary plexus.

**Supplemental Table 1.** Baseline characteristics of male participants in the study.

|  | | | | | | | | | | | | | | |  |
| --- | --- | --- | --- | --- | --- | --- | --- | --- | --- | --- | --- | --- | --- | --- | --- |
|  | |  | | Age group (years) | | | | | | | | | *P* | |  |
|  | | Total (n=449) | | 20–29 | | 30–39 | | 40–49 | | 50–59 | ≥ 60 | |  |  |  |
|  |  |  |  | (n=32) | | (n=229) | | (n=90) | | (n=70) | (n=28) | |  |  |  |
| Age (mean ± SD, years) | | 40.9 ± 10.3 | | 27.1 ± 1.9 | | 34.6 ± 2.5 | | 44.6 ± 2.9 | | 53.1 ± 2.5 | 65.6 ± 4.1 | | <0.001 | |  |
| Education level, n (%) | | | |  | |  | |  | |  |  | | <0.001 | |  |
| Middle school and below | | 94 (20.9） | | 3 (9.4) | | 16 (7.0) | | 24 (26.7) | | 32 (45.7) | 19 (67.9) | |  | |  |
| College / University | | 352 (78.4) | | 29 (90.6) | | 210 (91.7) | | 66 (73.3) | | 38 (54.3) | 9 (32.1) | |  | |  |
| Current smoking, n (%) | | 199 (44.3) | | 15 (46.9) | | 96 (41.9) | | 51 (56.7) | | 30 (42.9) | 7 (25.0) | | 0.03 | |  |
| Current drinking, n (%) | | 123 (27.4) | | 6 (18.8) | | 52 (22.7) | | 38 (42.2) | | 22 (31.4) | 5 (17.9) | | 0.004 | |  |
| BMI (mean ± SD, kg/m^2^) | | 25.1 ± 3.3 | | 25.2 ± 4.2 | | 25.0 ± 3.3 | | 25.8 ± 3.6 | | 24.9 ± 2.2 | 24.0 ± 2.3 | | 0.09 | |  |
| MAP (mean ± SD, mmHg) | | 91.1 ± 7.3 | | 91.8 ± 6.7 | | 90 ± 7.1 | | 92.5 ± 7.2 | | 92.4 ± 7.3 | 91.3 ± 8.8 | | 0.03 | |  |
| SE (mean ± SD, D) | | −1.2 ± 1.6 | | −1.6 ± 1.6 | | −1.7 ± 1.5 | | −1.0 ± 1.4 | | −0.5 ± 1.3 | 0.8 ± 1.1 | | <0.001 | |  |
| AL (mean ± SD, mm) | | 24.1 ± 0.6 | | 24.2 ± 0.4 | | 24.2 ± 0.6 | | 24.0 ± 0.5 | | 23.9 ± 0.6 | 23.7 ± 0.4 | | <0.001 | |  |
| SSI (mean ± SD) | | 8.6 ± 0.6 | | 8.5 ± 0.8 | | 8.6 ± 0.6 | | 8.7 ± 0.6 | | 8.5 ± 0.6 | 8.3 ± 0.8 | | 0.04 | |  |
| SD = standard deviation; BMI = body mass index; MAP = mean arterial pressure; SE = spherical equivalent refraction; D = diopter; AL = axial length; SSI = signal strength index of optical coherence tomography angiography image | | | | | | | | | | | | | | |  |
|  |  |  |  |  |  |  |  |  |  |  |  |  |  |  |  |
|  | | | | | | | | | | | | | | |  |
| **Supplemental Table 2.** Baseline characteristics of female participants in the study. | | | | | | | | | | | | | | | |
|  |  | | Age group (years) | | | | | | | | | | | *P* | |
|  | Total (n=578) | | 20–29 | | 30–39 | | 40–49 | | 50–59 | | | ≥ 60 | |  |  |
|  |  |  | (n=47) | | (n=287) | | (n=150) | | (n=83) | | | (n=20) | |  |  |
| Age (mean ± SD, years) | 40.0 ± 9.3 | | 26.9 ± 1.9 | | 34.3 ± 2.6 | | 44.2 ± 2.9 | | 53.4 ± 2.6 | | | 64.7 ± 4.1 | | <0.001 | |
| Education level, n (%) | | |  | |  | |  | |  | | |  | | <0.001 | |
| Middle school and below | 120 (20.4） | | 2 (4.3) | | 19 (6.6) | | 44 (29.3) | | 38 (45.8) | | | 17 (85.0) | |  | |
| College / University | 461 (78.5) | | 45 (95.7) | | 263 (91.6) | | 106 (70.7) | | 44 (53.0) | | | 3 (15.0) | |  | |
| Current smoking, n (%) | 3 (0.5) | | 1 (2.1) | | 0 (0) | | 1 (0.7) | | 1 (1.2) | | | 0 (0) | | 0.31 | |
| Current drinking, n (%) | 1 (0.2) | | 0 (0) | | 1 (0.3) | | 0 (0) | | 0 (0) | | | 0 (0) | | 0.90 | |
| BMI (mean ± SD, kg/m^2^) | 22.7 ± 3.0 | | 21.8 ± 3.6 | | 22.5 ± 3.0 | | 23.2 ± 2.7 | | 22.9 ± 2.7 | | | 24.1 ± 3.0 | | 0.007 | |
| MAP (mean ± SD, mmHg) | 84.8 ± 9.1 | | 83.7 ± 7.9 | | 83.1 ± 8.5 | | 86.7 ± 9.4 | | 86.6 ± 10.1 | | | 88.2 ± 9.2 | | <0.001 | |
| SE (mean ± SD, D) | −1.6 ± 1.7 | | −1.7 ± 1.5 | | −2.1 ± 1.6 | | −1.5 ± 1.7 | | −0.6 ± 1.4 | | | 0.1 ± 1.1 | | <0.001 | |
| AL (mean ± SD, mm) | 23.9 ± 0.6 | | 24.0 ± 0.5 | | 24.0 ± 0.6 | | 23.8 ± 0.6 | | 23.7 ± 0.5 | | | 23.6 ± 0.5 | | <0.001 | |
| SSI (mean ± SD) | 8.6 ± 0.6 | | 8.6 ± 0.6 | | 8.5 ± 0.6 | | 8.6 ± 0.6 | | 8.7 ± 0.5 | | | 8.2 ± 0.7 | | 0.006 | |
| SD = standard deviation; BMI = body mass index; MAP = mean arterial pressure; SE = spherical equivalent refraction; D = diopter; AL = axial length; SSI = signal strength index of optical coherence tomography angiography image | | | | | | | | | | | | | | | |
|  |  |  |  |  |  |  |  |  |  |  |  |  |  |  |  |

| **Supplementary Table 3.** Vessel densities of the whole measured area, parafovea, and four quadrants in the SCP and DCP stratified by age and sex. | | | | | | | | | | | | | | |
| --- | --- | --- | --- | --- | --- | --- | --- | --- | --- | --- | --- | --- | --- | --- |
|  |  | SCP (%) | | | | | |  | DCP (%) | | | | | |
|  |  | Whole | Parafovea | Temporal | Superior | Nasal | Inferior |  | Whole | Parafovea | Temporal | Superior | Nasal | Inferior |
| Male and Female | 20–29 | 47.1 ± 2.3 | 50.9 ± 2.7 | 49.2 ± 2.7 | 52.6 ± 3.2 | 50.1 ± 3.2 | 51.9 ± 3.0 |  | 51.7 ± 2.9 | 54.3 ± 3.3 | 54.5 ± 2.9 | 54.0 ± 4.1 | 54.8 ± 3.0 | 53.6 ± 3.8 |
|  | 30–39 | 47.0 ± 2.3 | 50.9 ± 2.5 | 49.1 ± 2.6 | 52.8 ± 2.7 | 49.9 ± 2.9 | 51.9 ± 3.0 |  | 51.7 ± 2.6 | 54.4 ± 2.9 | 54.5 ± 2.8 | 54.4 ± 3.2 | 54.8 ± 2.8 | 54.1 ± 3.4 |
|  | 40–49 | 46.9 ± 2.4 | 50.9 ± 2.5 | 49.0 ± 2.7 | 52.6 ± 2.6 | 50.0 ± 2.8 | 52.0 ± 2.8 |  | 51.1 ± 2.5 | 53.9 ± 2.7 | 54.0 ± 2.8 | 53.7 ± 3.1 | 54.3 ± 2.7 | 53.7 ± 3.2 |
|  | 50–59 | 45.8 ± 2.6 | 49.6 ± 2.7 | 48.0 ± 2.9 | 51.2 ± 3.2 | 48.9 ± 2.7 | 50.5 ± 3.2 | | 50.1 ± 2.7 | 52.7 ± 2.9 | 52.8 ± 2.8 | 52.6 ± 3.4 | 52.9 ± 3.1 | 52.4 ± 3.4 |
|  | ≥ 60 | 45.3 ± 2.6 | 49.2 ± 2.9 | 47.4 ± 4.0 | 50.9 ± 3.0 | 48.4 ± 3.0 | 50.1 ± 3.3 | | 49.0 ± 2.4 | 51.7 ± 2.8 | 51.8 ± 3.1 | 51.6 ± 3.3 | 52.2 ± 2.9 | 51.3 ± 3.2 |
|  |  |  |  |  |  |  |  | |  |  |  |  |  |  |
| Male | 20–29 | 46.3 ± 2.8 | 49.9 ± 3.4 | 48.4 ± 2.9 | 51.6 ± 4.1 | 49.1 ± 4.2 | 50.6 ± 3.5 | | 50.0 ± 2.5 | 52.3 ± 2.9 | 52.4 ± 2.7 | 52.4 ± 3.9 | 52.9 ± 2.7 | 51.7 ± 3.4 |
|  | 30–39 | 47.1 ± 2.4 | 50.8 ± 2.5 | 49.0 ± 2.6 | 52.7 ± 2.7 | 49.9 ± 2.9 | 51.7 ± 3.0 |  | 50.7 ± 2.6 | 53.1 ± 2.7 | 53.2 ± 2.6 | 53.0 ± 3.0 | 53.5 ± 2.7 | 52.5 ± 3.2 |
|  | 40–49 | 47.0 ± 2.4 | 50.8 ± 2.5 | 48.7 ± 2.5 | 52.3 ± 2.7 | 50.0 ± 2.9 | 52.1 ± 3.0 |  | 50.4 ± 2.4 | 52.8 ± 2.5 | 53.0 ± 2.6 | 52.6 ± 3.0 | 53.2 ± 2.6 | 52.5 ± 2.9 |
|  | 50–59 | 45.6 ± 2.7 | 49.2 ± 2.9 | 47.4 ± 3.1 | 50.6 ± 3.7 | 48.7 ± 2.7 | 50.0 ± 3.5 |  | 49.2 ± 2.9 | 51.5 ± 3.1 | 51.8 ± 3.1 | 51.4 ± 3.7 | 51.8 ± 3.3 | 51.1 ± 3.5 |
|  | ≥ 60 | 45.2 ± 3.0 | 49.0 ± 3.2 | 46.9 ± 4.8 | 50.5 ± 3.2 | 48.3 ± 3.3 | 50.5 ± 3.0 |  | 48.1 ± 2.2 | 50.7 ± 2.4 | 50.5 ± 3.0 | 50.4 ± 2.9 | 51.1 ± 2.7 | 50.6 ± 2.7 |
|  |  |  |  |  |  |  |  |  |  |  |  |  |  |  |
| Female | 20–29 | 47.7 ± 1.8 | 51.6 ± 2.0 | 49.7 ± 2.5 | 53.3 ± 2.1 | 50.8 ± 2.1 | 52.8 ± 2.3 | | 52.8 ± 2.5 | 55.6 ± 2.8 | 55.9 ± 2.1 | 55.2 ± 3.8 | 56.2 ± 2.4 | 55.0 ± 3.5 |
|  | 30–39 | 46.9 ± 2.3 | 51.0 ± 2.5 | 49.2 ± 2.6 | 52.9 ± 2.7 | 49.8 ± 2.9 | 52.1 ± 3.0 |  | 52.5 ± 2.4 | 55.5 ± 2.5 | 55.6 ± 2.5 | 55.4 ± 3.0 | 55.8 ± 2.5 | 55.4 ± 3.0 |
|  | 40–49 | 46.9 ± 2.4 | 51.0 ± 2.5 | 49.1 ± 2.8 | 52.8 ± 2.6 | 50.0 ± 2.7 | 52.0 ± 2.7 |  | 51.6 ± 2.6 | 54.5 ± 2.7 | 54.6 ± 2.8 | 54.3 ± 3.0 | 55.0 ± 2.6 | 54.3 ± 3.1 |
|  | 50–59 | 46.1 ± 2.4 | 50.0 ± 2.5 | 48.5 ± 2.6 | 51.7 ± 2.6 | 49.1 ± 2.8 | 50.9 ± 2.9 | | 50.8 ± 2.2 | 53.7 ± 2.4 | 53.7 ± 2.2 | 53.7 ± 2.8 | 53.9 ± 2.5 | 53.4 ± 2.9 |
|  | ≥ 60 | 45.4 ± 2.0 | 49.4 ± 2.5 | 48.1 ± 2.4 | 51.5 ± 2.8 | 48.5 ± 2.7 | 49.6 ± 3.7 | | 50.3 ± 2.3 | 53.2 ± 2.7 | 53.6 ± 2.3 | 53.2 ± 3.1 | 53.7 ± 2.6 | 52.3 ± 3.6 |
| SCP = superficial retinal capillary plexus; DCP = deep retinal capillary plexus | | | | | | | | | | | | | | |
